# Pointing to Visible and Invisible Targets

**DOI:** 10.1007/s10919-017-0270-3

**Published:** 2018-01-11

**Authors:** Zoe M. Flack, Martha Naylor, David A. Leavens

**Affiliations:** School of Psychology, University of Sussex, Falmer, East Sussex, BN1 9QH UK

**Keywords:** Pointing, Deictic gestures, Nonverbal communication, Paralinguistic gestures

## Abstract

We investigated how the visibility of targets influenced the type of point used to provide directions. In Study 1, we asked 605 passersby in three localities for directions to well-known local landmarks. When that landmark was in plain view behind the requester, most respondents pointed with their index fingers, and few respondents pointed more than once. In contrast, when the landmark was not in view, respondents pointed initially with their index fingers, but often elaborated with a whole-hand point. In Study 2, we covertly filmed the responses from 157 passersby we approached for directions, capturing both verbal and gestural responses. As in Study 1, few respondents produced more than one gesture when the target was in plain view and initial points were most likely to be index finger points. Thus, in a Western geographical context in which pointing with the index finger is the dominant form of pointing, a slight change in circumstances elicited a preference for pointing with the whole hand when it was the second or third manual gesture in a sequence.

Humans use a diverse array of deictic gestures, from pointing with the lips (Enfield 2001), to index-finger pointing (Eibl-Eibesfeldt 1989), to pointing with the whole hand (e.g. Wilkins 2003). Even within a culture, people display remarkable variety in their pointing hand shapes (Kendon and Versante 2003), with different shapes used for different functions. Kendon and Versante (2003) analyzed the pointing gestures of Neapolitans, finding that different hand shapes signified different dialectical functions. Similarly, Wilkins (2003) noted that the Arrernte people of Australia frequently give directions with whole-hand pointing gestures. Enfield et al. (2007) analyzed the variety of pragmatic functions of different pointing types among Lao speakers in Laos, finding that “big” points signified a primary location marker, whereas another, “small” form of pointing was used to more subtly support location information provided in speech. Hence, research over the last couple of decades has revealed pointing with the index finger to be—far from the “canonical” human pointing gesture—merely one of a large number of gestural devices for deixis that humans use, often as paralinguistic adjuncts to ongoing discourse, and variable in both form and semantic function within and between cultures (e.g. Enfield 2001; McNeill 2003).

Variations in gesture frequency, form, and size can reflect multiple communicative demands in a conversational interaction, although there is evidence that humans continue to display some gestures, even when not visible by the recipient (Alibali et al. 2001), the speaker’s awareness of the recipient’s knowledge is important. Cleret de Langavant et al. (2011) found differences in pointing and underlying neural activity when participants pointed for a recipient rather than pointed for no one in particular, demonstrating significant cerebral blood flow changes in the right hemisphere during communicative pointing, compared to non-communicative pointing. Elsewhere, use of less elaborated gestures when participants had a shared understanding of a communicative topic suggests that speakers use different communicative tactics depending on the different task demands presented by varying degrees of shared knowledge between sender and receiver (Gerwing and Bavelas 2004; Holler and Stevens 2007).

In experimental studies, the number of digits extended by the pointing hand in humans is also subject to dynamic contextual influences. For example, Iverson and Goldin-Meadow (1997) reported that when they blindfolded otherwise sighted participants in their study, these people displayed a dramatic shift away from pointing with the index finger and towards pointing with the whole hand, when gesturing during a Piagetian conservation task. In this respect, they resembled congenitally blind children, who also tended to point with their whole hands. This finding was later replicated by Iverson and Goldin-Meadow (2001), who commented, “the fact that the blindfolded children also use [index-finger] pointing gestures infrequently, suggests that even temporary loss of vision affects the ability to establish a line of regard” (p. 420). Thus, simply blocking participants’ visual access has a dramatic effect on the number of fingers with which people pointed, in some contexts. Iverson and Goldin-Meadow (2001) interpreted these patterns to suggest that there were two contrasting cognitive tactics at play: where a target could be encompassed by a line of regard, then pointing with the index finger served to augment the visual perception of the referent, but where the referent could not be seen—e.g. when participants were either congenitally blind or sighted, but blindfolded—then participants used a communicative tactic focussed on path segments, as a series of waypoints to the referent. These findings led us to hypothesize that blocking visual access to a referent might also alter the shape of the pointing hand in more naturalistic, less controlled circumstances (i.e. to explore the ecological validity of the previous, laboratory-based findings). Here, we wanted to find out whether this change in the number of fingers extended in a pointing hand was merely an artifact of laboratory testing or a more general phenomenon. If we find that, for example, people in an outdoors, naturalistic setting also pointed more with the whole hand when direct sight of a referent was blocked, then this would be consistent with the interpretation of Iverson and Goldin-Meadow (2001) that establishing a line of regard is important to hand shapes while pointing. In contrast, if we fail to find this influence of target visibility in a more naturalistic context, then this might implicate other aspects of their experimental or laboratory environment than line of sight.

In a task eliciting directions to local landmarks, Iverson (1999) reported that “information about direction and location tended to be conveyed primarily in gesture” (p. 1140). Displacement is a defining feature of language (e.g. Fitch 2010), and with the present studies we sought to impose a problem of displaced reference. We expected to elicit substantial amounts of pointing behavior, providing a window into how visible and invisible targets influence the morphology of nonverbal referential signaling. Relative to situations in which the referent is clearly visible, we thought an invisible displacement condition would require greater gestural elaboration, and sought to directly test this assumption, by measuring gestural sequence lengths.

In Study 1, we adapted a procedure by Kita (2003), and administered two experimental conditions: in the In-view condition, a researcher asked passersby for directions to a local landmark that was fully in view behind the researcher. In the Out-of-view condition, the same researcher asked passersby for directions to the same local landmark that was located at a similar or identical distance, but completely blocked from view by buildings. In each case, we recorded the palmar orientation and number of extended digits of the pointing hand, if any, for each pointing gesture displayed in this study. We administered this protocol in three different locations: on an English university campus, in a large English city, and in a small English town. We expected to see longer gesture sequences in the Out-of-view condition than the In-view condition because of the need to impart more information in the absence of a visible target; that is, we expected that the invisible target would create a more demanding communicative task, as evidenced by gesture sequence length. We also expected there would be more whole-handed pointing in the Out-of-view condition than in the In-view condition, based on the findings of Iverson and Goldin-Meadow (1997, 2001).

In Study 2, we used a similar procedure to that of Study 1, but used concealed recording equipment to capture the naturally occurring speech and gesture of our participants. We covertly collected audio and video recordings and obtained consent “after the fact”. With this study, we aimed to (a) confirm the findings of Study 1, (b) examine speech-gesture relationships, and (c) examine palm orientations while pointing (Kendon and Versante 2003).

## Study 1

### Method

#### Participants

Data were collected from 605 participants; 200 in the city of Brighton (100 in the In-view condition of which 48 were females, and 100 in the Out-of-view condition of which 56 were females), 205 in the town of Devizes (100 in the In-view condition of which 63 were females and 105 in the Out-of-view condition of which 52 were females), and finally, 200 on a university campus in the south of England (100 in the In-view condition of which 53 were females and 100 in the Out-of-view condition of which 54 were females). Subjects were adults who were approached in one of the three locations and assigned to a condition based on their proximity to the target location. There were no exclusion criteria for selection of participants, and the ethnic composition of each sample was apparently representative of each locale, although there was no systematic collection of data on ethnicity.

#### Locations and Targets

In Brighton, the target location was the Royal Pavilion. The location of the researcher was equidistant from the target location in both the Out-of-view and In-view conditions, a distance of 198 m. In Devizes, the target location was a local public library; here, the distance of the researcher from the target location was 116 m in the Out-of-view condition and 100 m in the In-view condition. At the university campus, the target was the main library building and the researcher was equidistant from this landmark in the two conditions, at a distance of 152 m.

#### Procedure

Participants were approached in close proximity to the target in one of the three locations. There were two conditions for each location; one where the target was in view of the participant (the In-view condition) and another where the target was not in view of the participant (the Out-of-view condition). In the In-view condition the target was directly in front of the participant (i.e. directly behind the researcher), and in the Out-of-view condition the target was not in direct view, due to intervening buildings, although a simple right-angle path could be described to the target. Using a standardized script, participants were asked for directions to the target location. Participants were always approached when they were already facing towards the target landmarks, so that the researcher had her back to the target location. Their pointing gestures were recorded on a paper sheet by the researcher who was both observer and interlocutor for every interaction, and observation ended when the participants withdrew from the interaction.

#### Behavioral Measures

Five types of pointing gestures were initially recorded, categorized by the position of the forearm, hand, and fingers, following Kendon and Versante (2003). In this coding scheme, there were two kinds of index-finger points: (a) *index palm down* (ID) where the forearm was pronated, palm facing downwards and index-finger extended and (b) *index palm vertical* (IV) where the forearm was extended in a neutral position, the palm of the hand in a vertical position and index-finger extended. There were three types of open-hand points: (c) *whole*-*hand palm up* (OU) where the hand was fully open with palm supine, (d) *whole*-*hand oblique* (OB) had the palm at an oblique angle and (e) *whole*-*hand palm vertical* (OV). As reported, below, however, interobserver reliability for this five-category coding scheme was poor, therefore, categories were collapsed into two categories for analysis, here: index-finger points and whole-hand points.

#### Reliability

Reliability was assessed by observer and by order of gesture (i.e. first, second, and third gestures). In each of the three locations, 30 interactions (90 in total) were independently coded by two observers, and assessed for interobserver reliability (15% of observations). In both Brighton and the university campus, the same two observers were used, so reliability was assessed on the 60 cases coded by these two individuals, and reliability is reported separately for the Devizes location, for an additional 30 cases. In the reliability samples, we examined (a) the agreement between two observers that a first, second and third gesture occurred, and (b) given that two observers agreed that a pointing gesture occurred, the agreement on the type of pointing gesture.

Our initial coding of gestures yielded Cohen’s kappa values ranging from .44 to .73. When we collapsed the data into two types: index-finger and whole-hand pointing, reliability estimates significantly improved, and therefore we focus on these two types of gesture in our analyses. In all three locations, there was 100% agreement on whether a point occurred as the first gesture, 100 or 93% (Cohen’s kappa = .86) agreement on whether a second gesture occurred, and 90% (Cohen’s kappa = .45) or 97% agreement on whether a third gesture occurred (in Devizes, both observers agreed in 29 out of 30 cases that no third gesture occurred, and there was one disagreement about the presence of a third gesture, hence kappa is not appropriate). For the first gesture, there was 100% agreement on which type of gesture, agreement on type of gesture was between 87% (Cohen’s kappa = .72) and 100%. Because there were only 3 cases in which both observers agreed that a third gesture occurred, we did not include third gestures in analyses related to gesture type or the effects of visibility on gesture type.

### Results

#### Initial Analyses

There were no effects of location (i.e. whether the data were collected in Brighton, the university campus, or Devizes) or gender of participant on either gesture sequence lengths or gesture types, therefore neither location nor gender will be further considered. Of the 605 participants, one did not display a manual pointing gesture (.17%), hence the total sample size in the following analyses is 604.

#### Sequence Length

**Fig. 1:**
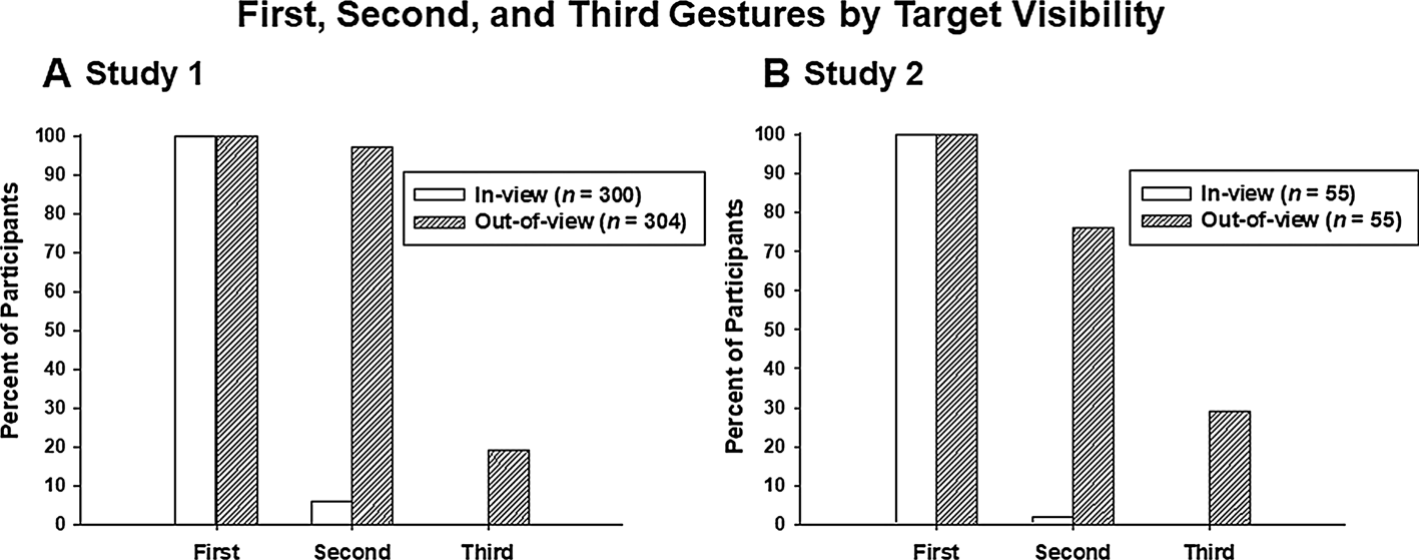
The percentage of participants who displayed first, second, and third manual pointing gestures, by condition. **a** Study 1, **b** study 2

Sequences ranged in length from 1 to 3 gestures (no participant displayed more than 3 pointing gestures). Unsurprisingly, gesture sequence length was significantly longer in the Out-of-view condition (Mdn = 2 gestures) than in the In-view condition (Mdn = 1 gesture); *U*(1) = 3608, *Z* = − 21.77, *p* < .001. As depicted in Fig. 1a, 97% (296/304) of participants approached in the Out-of-view condition went on to display a second gesture, whereas only 6% (17/300) of participants in the In-view condition did so (χ^2^(1, *N* = 604) = 505.57, *p* < .001). Nineteen percent (57/304) of the participants in the Out-of-view condition displayed a third point, whereas none of the 300 participants in the In-view conditions did so (χ^2^(1, *N* = 604) = 62.11, *p* < .001). Typically, participants in the Out-of-view condition used subsequent points, after their first, to outline a route to the landmark in question.

#### Effects of Target Visibility on Gesture Type

**Fig. 2:**
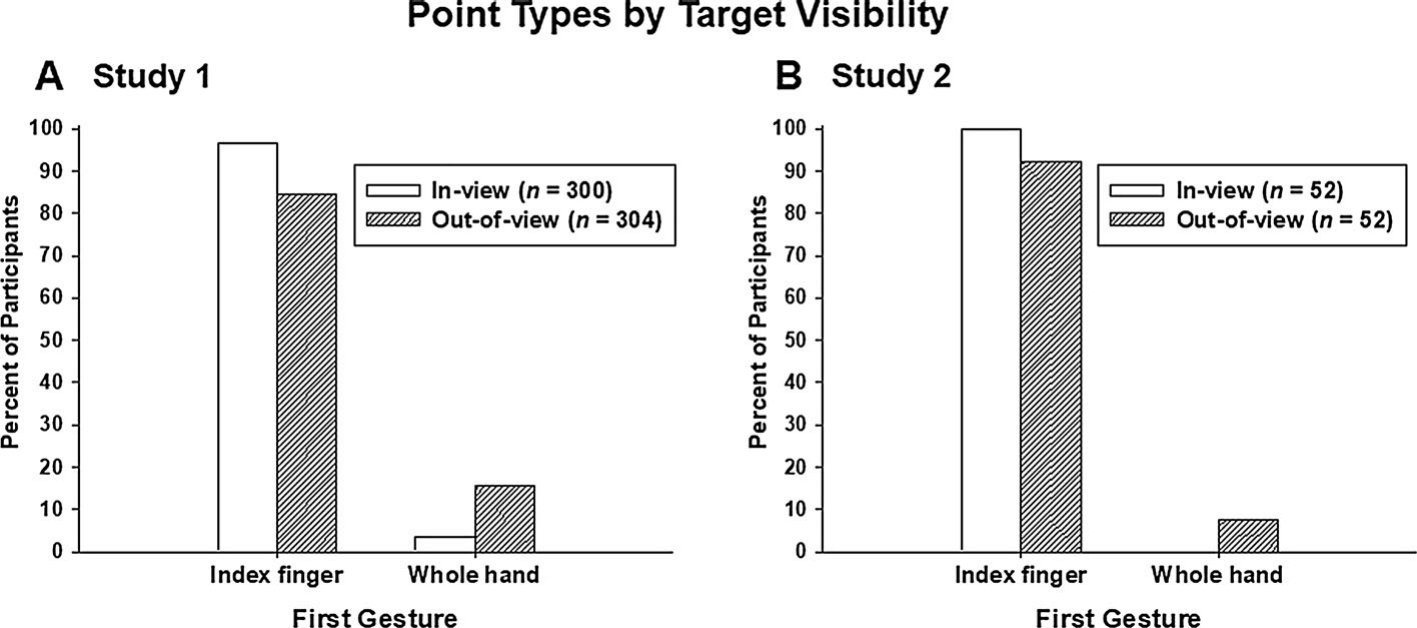
The percentage of participants who displayed points of different types (index-finger, whole-hand). **a** Study 1, **b** study 2

There were significant effects of target visibility on gesture type for both the first gesture (χ^2^(1, *N* = 604) = 25.98, *p* < .001) and the second gesture (χ^2^(1, *N* = 313) = 4.85, *p* = .028); see Fig. 2a. There were substantially more whole-handed points displayed in the Out-of-view condition, compared to when the targets were in full view (the In-view condition).

#### Use of Whole-Hand Pointing in the Out-of-View Condition

No participant in the In-view condition displayed more than two manual points. In the Out-of-view condition, 57 participants displayed three consecutive points. With increasing ordinal number of the pointing gesture, there was an increased probability that a point with the whole hand would be displayed (Cochran’s *Q*(2) = 43.45, *p* < .001).

#### Indexicality Indices

**Fig. 3:**
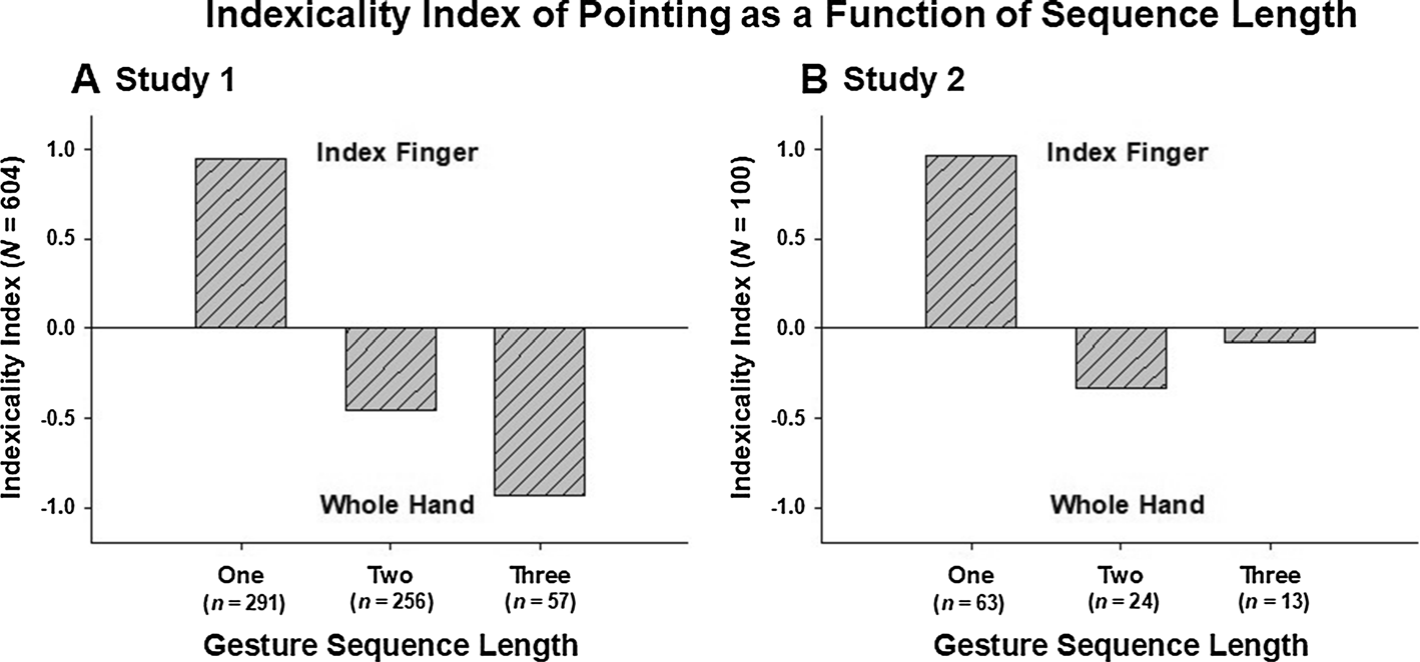
Indexicality indices as a function of gesture sequence length. Positive bars indicate predominance of index-finger points, negative bars indicate a preponderance of whole-hand points. **a** Study 1, **b** study 2

Given the large number of points with the whole hand recorded during this study, here we categorized people in terms of the degree to which they displayed index-finger or whole-hand pointing, as a function of the length of their gestural sequences. We depict these data with an “indexicality index”, defined in Leavens and Hopkins (1999) as:

$$\frac{I - W}{I + W}$$

where *I* means the frequency of index-finger points and *W* means the frequency of whole-hand points; this renders a scale ranging from − 1.0 to + 1.0, with positive numbers for samples in which index-finger points outnumber whole-hand points and negative numbers for the opposite result. In the case that *I* = *W*, zero is assigned as the quotient. As is evident in Fig. 3a, there is an immense swing away from a preference for pointing with the index finger to pointing with the whole hand, with an increase in subjects’ gestural sequence lengths. Thus, pointing with the whole hand became more prominent in these samples’ gestural repertoires as their apparent need to elaborate increased.

#### Sequences

We characterized two-gesture sequences for both conditions as I–I, I–W, W–I, or W–W where W refers to whole hand point, and I refers to an index finger point. There was a significant difference in the distributions of these two-gesture sequences across the In-view (*n* = 17) and Out-of-View (*n* = 239) communicative contexts (χ^2^(3, *N* = 256) = 11.81, *p* = .008). Overall, 70 of 256 people (27.3%) who displayed two-gesture sequences displayed two successive index-finger points, avoiding use of the whole hand, but the majority of people displaying two-gesture sequences incorporated at least one whole-hand point into their sequences (186/256 or 72.7%). This, despite the fact that the number of two-gesture sequences beginning with an index-finger point (213/256 or 83.2%) was significantly larger than the number of two-gesture sequences beginning with a whole-hand point (43/256 or 16.8%; binomial test, *Z*(255) = 10.56, *p* < .001). Hence, although pointing with the index finger was the preferred initial gesture, most people who felt the need to display two gestures in this observational context incorporated a whole-hand point into the sequence.

### Discussion

We found evidence that context influences gesture production. Specifically, adults produced fewer gestures when the target location was visible than when it was not, consistent with our expectation that a single point to a visible target requires less elaboration. We also found fewer whole hand points when the target location was visible than when it was not. Index finger points accounted for more first gestures than other point types, but where further gestures were needed, these were more likely to be whole hand points.

Although we found evidence of contextual influences on gesturing we did not record speech. A reviewer of our initial submission recommended filming some additional trials because there is evidence that gestural responses vary as part of a wider communicative interaction (e.g. Enfield et al. 2007; McNeill 2003). Therefore, we were keen to examine the speech types accompanying these gestures, to address this possibility. Moreover, we achieved poor interobserver agreement in Study 1 on palm orientations, and it was expected that video records would foster better reliability on this measure, permitting comparison with Kendon and Versante (2003). Finally, as also noted by an anonymous reviewer, a second study with video records would permit a direct verification of the results of Study 1.

## Study 2

We obtained ethical approval for covert collection of audio and video recordings of the interactions in question with a proviso that consent be obtained “after the fact”. In Study 2 we used a similar procedure to that of Study 1, but used concealed recording equipment to capture the naturally occurring speech and gesture of our participants. The amount of time involved in setting up the covert filming apparatus and, especially, in obtaining post hoc informed consent, resulted in a reduced sample size, relative to Study 1. We aimed to confirm the patterns we found in Study 1, to examine the relationship between the content of speech and pointing types, and to increase interobserver reliability for the palm orientations during pointing.

### Method

#### Participants

Data were covertly collected from 157 adult passersby in one of two locations on a university campus in southeast England. Solitary adults walking toward the target location were approached and asked for directions to the library. Of those approached, 139 subsequently provided informed consent for the use of their video and audio recordings and became participants in the study, although we subsequently received one email request to withdraw. Following a change in the Out-of-view location, 14 cases were removed from analysis. An additional 11 trials in which we failed to adequately capture either the gesture or speech were removed, leaving data from 113 participants (67 females and 46 males), with declared ages ranging from 18 to 67 years (*M* = 23.46 years, *SD* = 8.92) available for analysis. As in Study 1, there were no exclusion criteria and ethnic makeup was broadly representative of the university population (i.e. highly multinational).

#### Locations and Targets

The target location in both conditions was the university’s library. Two additional locations, in which it was possible for discreet recording of the interaction to take place, were selected to provide the In-view and Out-of-view conditions. For the In-view condition the researcher was situated approximately 133 m from the library building, facing away from the library, so that passersby had a clear view of the library in the In-view condition. For the Out-of-view condition, the researcher was positioned approximately equidistant (~ 139 m) from the library building; in this location, the library was not visible due to intervening buildings, but participants were still facing the direction of the library to ensure similar body positioning and orientation for both conditions.

#### Materials and Equipment

A Sony digital high definition video camera recorder HDR-CS250 and a Sony ICD-UX532 audio recorder were used to collect the video and audio recordings. After the structured interaction, an information sheet was offered to all participants before they were asked to consent to their inclusion in the study.

#### Procedure

Experimenters took turns to be the actor and video camera operator. Before each interaction, experimenters captured a visual image of the numbered consent form. A handclap was recorded, enabling subsequent synchronization of the separate video and audio recordings. The actor concealed the audio recorder in their clothing before approaching subjects, asking for directions to the target destination using a scripted question: “Excuse me, I’m looking for the library, can you help at all?”

When the subject had completed their verbal and gestural response, the actor disclosed the true purpose of the interaction, explained that video and audio recordings had been made, and provided the information letter and consent form. Experimenters deleted recordings if subjects did not provide consent, although in all cases—except the one instance of delayed withdrawal from the study—subjects were only unable to consent due to personal time constraints, and none of those approached requested immediate withdrawal from the study. Audio and video recordings were synchronized and trimmed using Windows Live Movie Maker.

#### Behavioural Measures

The number, hand used, and type of individual pointing gestures for each interaction were recorded. Pointing gestures were again categorized by hand and finger position similar to that used in Kendon and Versante (2003) and described above. As in Study 1, interobserver reliability for the five-category coding scheme which included palmar orientation proved to be poor, therefore, categories were collapsed into two categories for analysis: index-finger points and whole-hand points.

Accompanying speech was categorized as: (a) path description (route), (b) location-specific (library), (c) waypoints (as per Iverson 1999), (d) a combination of these, and (e) other types of speech. Path description was that which described a route in terms of directions; phrases such as “go this way”, or “take a left turn”. Location-specific speech included phrases specifically referring to the target location rather than the journey, such as “over there” or “behind this building”. Waypointing speech, in which a route is described not by cardinal or relative directions, but by specific features on the route, included comments such as “turn right where those guys are” or “you see the square”. The use of waypoints to describe the route was common (see also, Iverson 1999) in the Out-of-view condition and provided a possible “visible” alternative to the out-of-view target. Where a waypoint was mentioned these were also coded for whether they were visible to the pointer at the time of the gesture.

#### Reliability

A sample of 17 recordings (15%) was randomly selected, independently coded by a research assistant, who was blind to the hypotheses being tested, and assessed for interobserver reliability. Reliability was assessed for agreement between the observers that first, second, and third gestures took place, the type of gesture, palm orientation and the speech type accompanying the gesture.

Interobserver agreement on palm orientation was even poorer than in Study 1, despite the advantage of recordings from which to code, with Cohen’s kappas ranging from .29 to .41. For this reason, palm orientation was excluded from further analysis, and we used the same dichotomous coding scheme that we used in Study 1 (whole-hand and index-finger points), to facilitate direct comparisons.

Observers reached 100% agreement that a gesture was produced and on the number of points elicited in each case. Interobserver reliability for the type of point used in the first gesture was 89% (Cohen’s kappa = .66) which is considered good (Bakeman and Gottman 1997), and 100% for both second and third gestures.

Agreement for speech type accompanying gestures was 93% (Cohen’s kappa = .90) for first gestures, 100% for second gestures, and there were insufficient third gestures in the reliability sample to calculate interobserver reliability.

Agreement between observers when judging whether a point occurred, which type of point and which type of speech was excellent.

### Results

#### Initial Analyses

Every participant produced at least one manual gesture. A total of 174 gestures by 113 passersby were recorded, of which 35 were categorized as whole-hand points, 127 index-finger points and 12 other gestures; thus, index-finger points were the most commonly displayed. The presence of a gesture was coded as indeterminate in three instances so these three participants’ data have been excluded, leaving 110 for further analysis. Index finger points constituted 91% (100/110) and whole-hand points 4% (4/110) of first gestures; other gestures comprised 5% (6/100). Second gestures were predominantly whole-hand points (56%; 24/43) and index-finger points (33%; 14/43); other gestures comprised 11% (5/43) of second gestures. Forty-four percent (7/16) of third gestures were whole-hand points, and 56% (9/16) were index-finger points. No effects of participant gender on the type of speech or gesture or the number of gestures used were found, so participant gender is not considered further.

#### Sequence Length

Although the initiations of the interactions were scripted, no provision was made for experimentally controlling the interaction beyond the initial approach. For this reason, it seems likely that as gestural sequences become longer any effects of target visibility will be harder to detect, and indeed less confidently attributed to the manipulations. Although 5 fourth gestures were observed, these were too few for analysis, so analysis was limited to the first two or three gestures—paralleling the analyses in Study 1—in all cases. The number of gestures (0–3) exhibited was significantly higher for approaches made by the first experimenter (*Mdn* = 2) than the second experimenter (*Mdn* = 1); Mann–Whitney *U*(1) = 1069.50, *z* = − 2.94, *p* = .001, *r* = − .28. We therefore analyzed every result separately by experimenter, but we found no differences between the two experimenters on any of the dependent measures except frequency; therefore, we combined all data across both experimenters for the analyses described below. As in Study 1, sequence length was significantly longer in the Out-of-view condition (*Mdn* = 2) than the In-view condition (*Mdn* = 1), (*U*(1) = 377, *z* = − 7.81, *p* < .001, *r* = − .74. As depicted in Fig. 1b, 76% (42/55) of participants approached in the Out-of-view condition went on to display a second gesture, whereas only 2% (1/55) of participants in the In-view condition did so, (*χ2*(1, *N* = 110) = 64.18, *p* < .001). Twenty-nine percent (16/55) of the participants in the Out-of-view displayed a third point whereas none of the 55 participants in the In-view condition did so, (*χ2*(1, *N* = 110) = 18.72, *p* < .001). Thus, those who could not see the target location produced more gestures than those with the target location in sight, which is the same pattern reported in Study 1 (compare Fig. 1a, b).

#### Effects of Target Visibility on Gesture Type

Pointing with either the index finger or the whole hand comprised 95% (104/110) of first gestures and 88% (38/43) of second gestures. Restricting analysis to only index-finger pointing and whole-hand pointing (i.e. ignoring “beckons” and “other gestures”) significantly reduced our power to discern associations between target visibility and gesture type, relative to Study 1. Nevertheless, we did find a qualitative similarity between the present study and Study 1: a significant effect of visibility on gesture type for the first gesture (*χ*2(1, *N* = 104) = 4.16, *p* < .041; however, please note that when this comparison is corrected for continuity, the effect is no longer statistically significant). Despite the low power of this analysis, the overall pattern is strikingly similar to that obtained for first gestures in Study 1, compare Fig. 2a with Fig. 2b). However, in the In-view condition, only one participant displayed a point as a second gesture, and no participants in the In-view condition displayed a third gesture, therefore it is not possible to test for an effect of target visibility on the second gesture types, as in Study 1, given the smaller sample.

As in Study 1, index-finger points were significantly more likely in both the In-view (binomial sign test, *p* < .001) and Out-of-view conditions (binomial sign test, *p* < .001) for first gestures (Fig. 2b). Also similar to Study 1, for second gestures, whole-hand points were the predominant gesture type (44%), rather than index-finger points (34%) for the Out-of-view condition (subsequent gestures were limited to a single case in the In-view condition so are not discussed further), although, due to the lower sample size, this was not a statistically significant difference in incidence of whole-hand points compared to index-finger points (binomial sign test, *p* = .14, *ns*).

#### Use of Whole-Hand Pointing in the Out-of-View Condition

Only 13 participants in the Out-of-view condition displayed a minimum sequence of three successive points, hence an equivalent analysis of point type in Study 2 is underpowered, relative to Study 1; nevertheless, this analysis revealed a statistical trend (Cochran’s *Q*(2, *N* = 13) = 5.17, *p* = .076) from a preponderance of index-finger pointing as first gestures to a preponderance of whole-hand pointing as third gestures). In a subsample of 34 individuals in the Out-of-view condition, who displayed two successive points, we found a significant change in gesture type from index-finger to whole-hand points (Wilcoxon signed ranks test, *Z*(33) = − 4.03, *p* < .001).

#### Indexicality Indices

Figure 3b depicts the swing from index-finger to whole-hand pointing in Study 2, confirming the general pattern observed in Study 1, albeit there is a lower magnitude of reliance on whole-hand pointing as third gestures, compared with Study 1. Sample size is 100, due to indeterminacy or non-pointing “other” first gestures in 10 cases.

#### Speech Type, Target Visibility, and Gesture Type

**Fig. 4:**
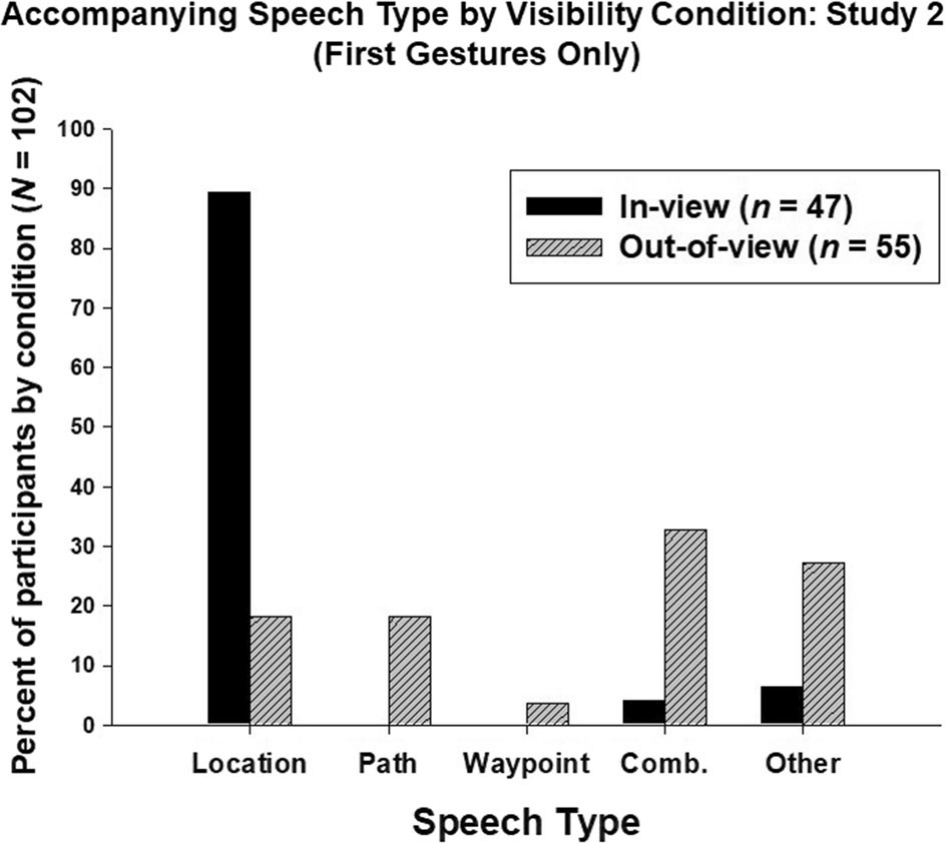
The percentage of participants, by condition, who displayed different kinds of accompanying speech with their first gestures. See text for speech types. “Comb.” = “Combination of speech types”

We considered “path description”, “location (library)”, “waypoint”, “combination”, “other”, and “no speech” categories in the following analyses. Eight of the 55 participants in the In-view condition displayed no accompanying speech with their first gestures, whereas none of the 55 participants in the Out-of-view condition failed to speak (binomial test, *p* = .008). Including the remaining 102 participants, there was a significant effect of viewing condition on the type of speech used for the first gesture (χ^2^(4, *N* = 102) = 50.64, *p* < .001). As is evident in Fig. 4, there were no verbal descriptions of paths or waypoints in the In-view condition, and significantly fewer verbal appeals to the target location (the library) in the Out-of-view condition, in relation to the In-view condition. There were insufficient subsequent gestures in the In-view condition to analyse second and third gestures. Individuals therefore used different verbal response types in the two locations: when the target was visible, they did not describe a route, and when the target was not visible, they were relatively less likely to verbalize the location of library with their first gestures, and adopted a more diverse range of verbal tactics in combination with their gestures.

Including 95 participants who pointed as their first gesture, there was no significant association between speech type and point type (index-finger vs. whole-hand; *χ*^*2*^(4, *n* = 95) = 3.01, *p* = .556). It seems, then, that target visibility influenced the type of speech used, but that point type and speech types were not significantly associated.

### Discussion

In Study 2 we again asked passersby to direct us to either a visible or non-visible target location, but this time we recorded these interactions on digital video. We found that, as in Study 1, gestural sequences were longer when the target location was not visible. We also found an effect of visibility of the target on the gesture type used, with whole-hand points being produced more frequently in the Out-of-view condition. Thus, the results of Study 2 broadly replicate the findings in Study 1 (see Figs. 1, 2 and 3) and, moreover, demonstrate an effect of target visibility on the types of speech accompanying pointing gestures.

#### General Discussion

In two naturalistic studies of pointing postures in 714 adults under two experimentally manipulated circumstances, the target was either (a) in view or (b) out of view. Remarkably, almost all passersby spontaneously pointed when faced with a stranger asking directions to a prominent local landmark. We hypothesized that barring visual access to even very large targets (local landmarks) might increase the proportion of points that were displayed with the whole hand. This turned out to be the case in both studies. However, first gestures were still, overall, primarily index-finger points. In both of these studies, pointing with the whole hand was increasingly displayed with the ordinal number of the gesture, which may reflect the subjects’ need to elaborate on their first gestures, particularly when the landmark was not visible. We also found greater gestural perseveration in the Out-of-view condition than the In-view condition. Thus, as predicted, barring immediate visual access to a landmark did elicit more pointing with the whole hand compared to a condition in which the target landmark was in full view. Our analysis of accompanying speech in Study 2 found no association between point and speech type, but we did find that participants used a much wider variety of speech types when their view of the target location was obscured. The proportion of index-finger points used was much lower when the target was not visible than when it was visible. Thus, our findings support the claim that establishing a line of regard is important to hand shapes and that manipulation of visual access affects pointing gestures (Iverson and Goldin-Meadow 2001).

In the present samples, sequences were overwhelmingly initiated with index-finger points. It therefore seems evident that in this geographical context, pointing with the index finger is the canonical first response. Wilkins (2003) claims some cultures do not use the index finger as the canonical pointing gesture, and provides evidence of cultures in which pointing with the lips is the canonical form of pointing. Therefore, we would be reluctant to claim generality of this finding beyond the geographical context in which our data were collected, viz., southern England.

Our data confirm previous observations by Kendon and Versante (2003), Wilkins (2003) and Haviland (2003) suggesting that pointing with the whole hand is a prominent component of the gestural repertoires of people from many parts of the world. For example, Haviland (2003) reported use of “the flat hand (with the palm held vertically, thumb side up, fingers grouped and extended outwards) to indicate vectors or directions” (p. 160). Kendon and Versante (2003) noted use of an index-finger point to denote a precise location, and a whole-hand point to indicate a more generalized location. Finally, Wilkins (2003) described two variants of whole-hand pointing in aboriginal Australians, the Arrernte, one of which is used to give cardinal directions. Our data demonstrate that pointing with the whole hand is a prominent gesture in southern England, as well, and the influence of target visibility on gesture form is not merely an artifact of laboratory testing.

We had expected that there might be a relationship between the type of pointing and the content of ongoing speech in this context, but this turned out not to be the case. However, we did find a significant relationship between speech content and the experimental context (In view vs. Out-of-view). Participants in the In-view condition overwhelming displayed landmark-centred speech, whereas participants in the Out-of-view condition, taken as a group, displayed more varied speech content. We found that the increased task demands of the Out-of-view condition elicited increased gestural and verbal elaboration. This finding adds to the debate about speech and gesture co-modulation in the cognitive sciences (i.e. the question of whether gesture and speech comprise a unitary system; for a review, see Hostetter and Alibali 2008), but does not resolve among different models of speech-gesture relationships.

We did not collect subject variable information, so we could not explore possible modulating effects of socioeconomic status, age, culture of origin, first language, or other possible factors. Moreover, we can add little to a growing scientific concern with the semiotic functions of different deictic gestures (see, e.g. Enfield 2001; Haviland 2003; Kendon and Versante 2003; Kita 2003; Wilkins 2003); this is, in part, because we had only two very specific observational contexts. We exerted no control over whether our participants were carrying items in their hands, and therefore did not address questions pertaining to the handedness of pointing.

While this is, to our knowledge, one of the largest studies of pointing hand shapes extant, we were unable to achieve acceptable levels of interobserver reliability on palm orientations during pointing. We struggled to assign only one palm orientation to each gesture due to the dynamic variability of the gestures we observed. This may be because we studied gestures produced by people momentarily stopped en route from one place to another, unlike Kendon and Versante (2003) who studied people who were seated in a café. We were therefore unable to make the detailed micropostural comparisons with previous work that we had initially planned. Many studies have distinguished pointing postures at the same level we did, so our results are comparable to a broad corpus of published work in this area, particularly with children (e.g. Blake et al. 1994; Franco and Butterworth 1996; Leung and Rheingold 1981). Overall, our interobserver reliability was very high, but our relatively poor reliability on palm orientation suggests that this aspect of hand posture while pointing merits further investigation; at present, we do not know why hand postures seemed to be more easily scored when people were at rest (as in Kendon and Versante 2003) than when briefly stopped while in motion (as in the present studies).

Because we did not systematically control the experimenter’s behavior as interactions progressed, we think that the findings for first gestures are more convincingly tied to visual access to local landmarks than the second or subsequent gestures. Specifically, there may have been systematic differences in the experimenter’s behavior after the first gestures in the two different conditions, whereas first gestures were elicited by scripted interactive protocols. For example, had the experimenter turned to look at a visible target after the first point in the In-view condition then this orienting response, rather than visual access, per se, may have suppressed additional pointing responses, and this warrants further investigation.

In conclusion, pointing with the whole hand is a prominent part of the nonverbal deictic repertoires of adults in the south and southwest of England, at least in response to a query about the direction of a local landmark. If that landmark is not in plain view, subjects elaborate their initial pointing gestures, usually integrating a whole-hand point into these elaborated sequences. Hence, manipulation of target visibility affects the number of fingers extended while pointing in our samples, comprising 717 human adults. Like many others (Blake et al. 1994; Clark 2003; Cochet and Vauclair 2010; Enfield et al. 2007; Haviland 2003; Hobaiter et al. 2014; Kendon and Versante 2003; Leavens and Hopkins 1999; Pika and Bugnyar 2011; Pika and Mitani 2006; Xitco et al. 2001), we suggest that increased attention to the full panoply of the forms of both human and nonhuman nonverbal deictic behaviors will reveal new insights into the psychology of nonverbal reference, which would be unattainable with too narrow a focus on one particular kind of pointing: pointing with the index finger.
